# Supplementary material for: Lunar farside volcanism 2.8 billion years ago from Chang’e-6 basalts
Source: Nature. 2024 Nov 15;643(8071):356–60. doi: 10.1038/s41586-024-08382-0 (PMC12240805; doi:10.1038/s41586-024-08382-0)
Supplement: Supplementary file 1 — All BSE images of 108 dated basalt fragments. [file 41586_2024_8382_MOESM1_ESM.pdf]

nature portfolio

https://doi.org/10.1038/s41586-024-08382-0

Supplementary information

Lunar farside volcanism 2.8 billion years ago from Chang'e-6 basalts

In the format provided by the authors and unedited

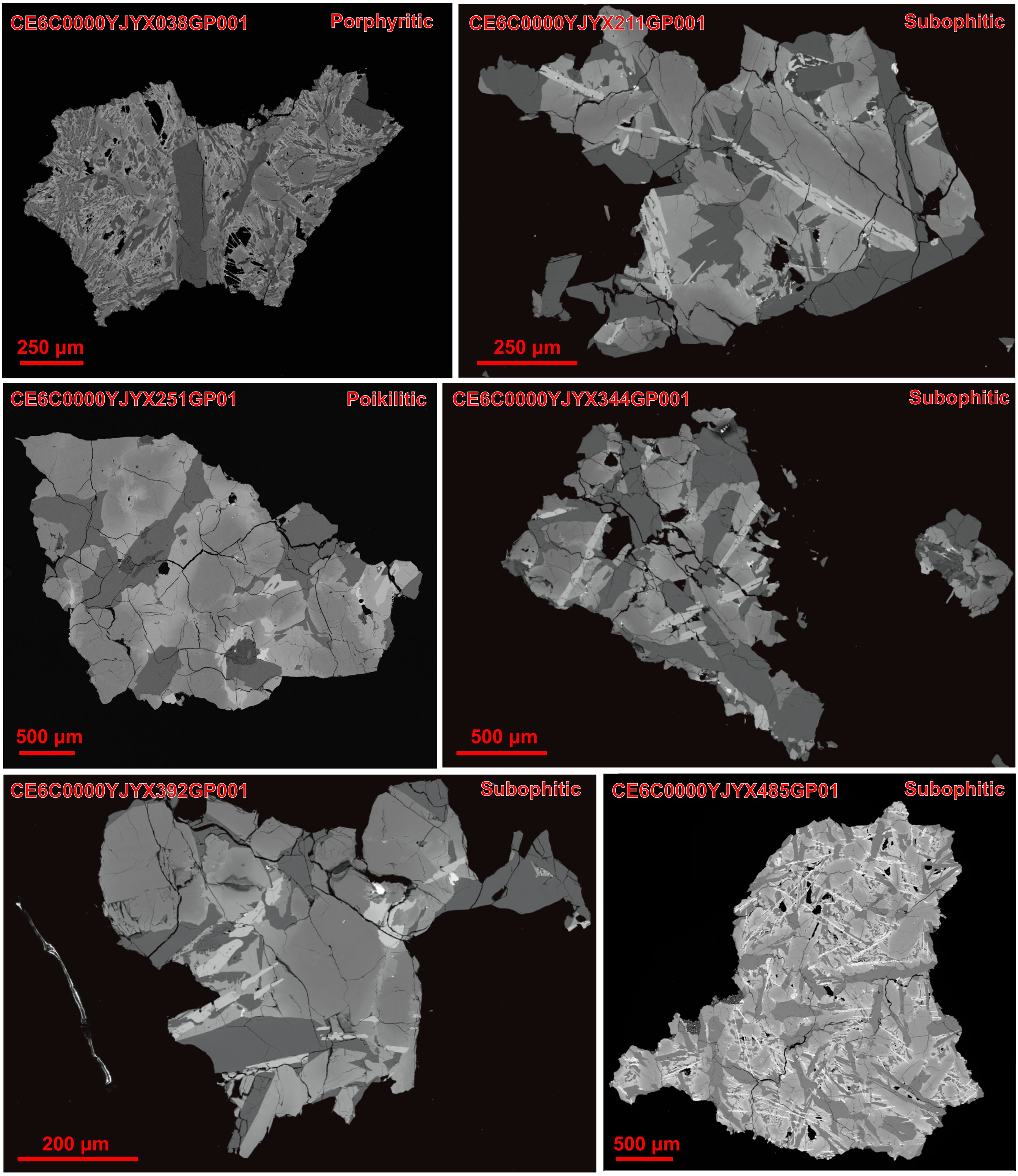
CE6C0000YJYX038GP001
Porphyritic
250 μm
CE6C0000YJYX211GP001
Subophitic
250 μm
CE6C0000YJYX251GP01
Poikilitic
500 μm
CE6C0000YJYX344GP001
Subophitic
500 μm
CE6C0000YJYX392GP001
Subophitic
200 μm
CE6C0000YJYX485GP01
Subophitic
500 μm

Fig. S1-1

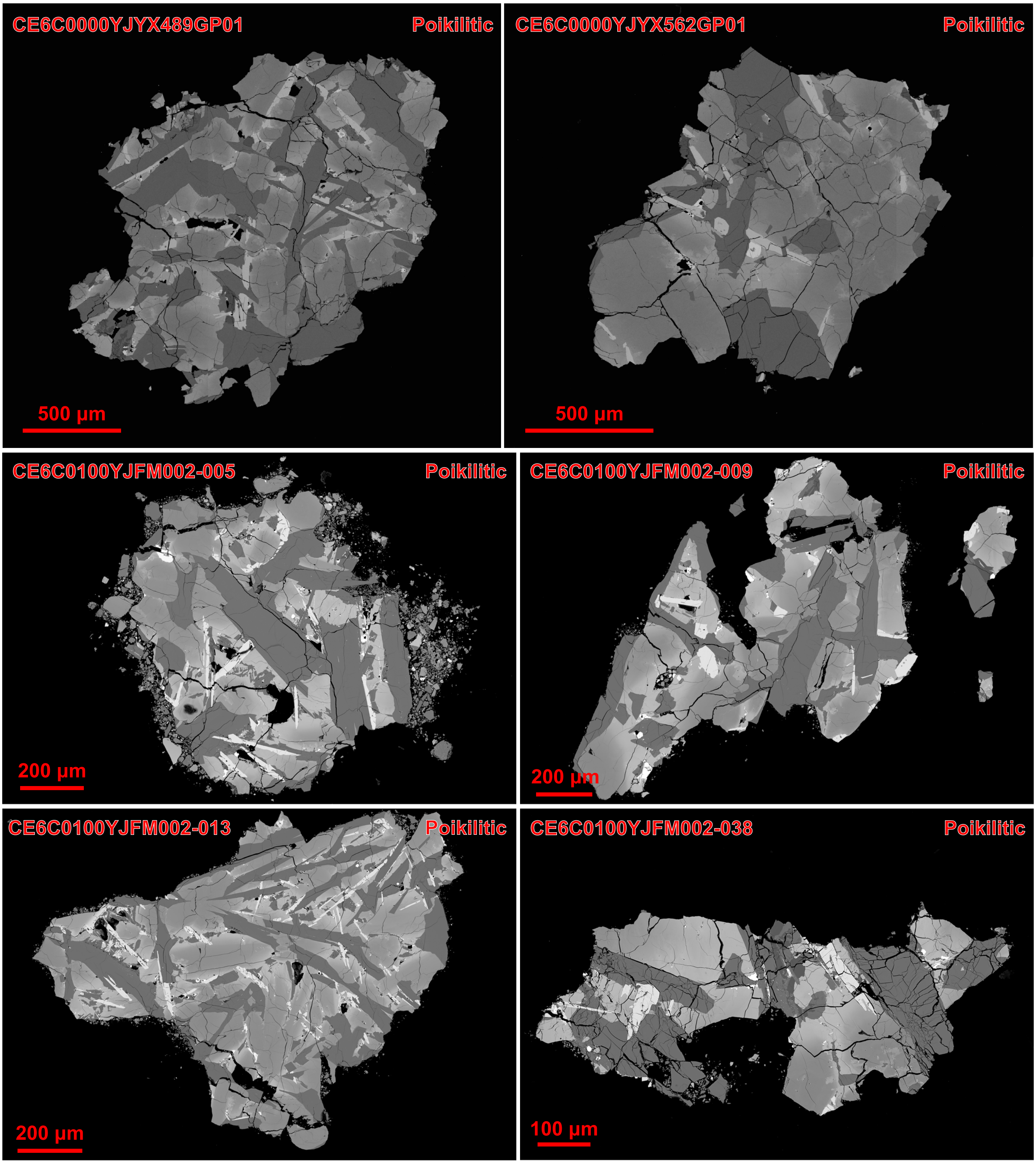

CE6C0000YJYX489GP01
Poikilitic
500 μm
CE6C0000YJYX562GP01
Poikilitic
500 μm
CE6C0100YJFM002-005
Poikilitic
200 μm
CE6C0100YJFM002-009
Poikilitic
200 μm
CE6C0100YJFM002-013
Poikilitic
200 μm
CE6C0100YJFM002-038
Poikilitic
100 μm

Fig. S1-2

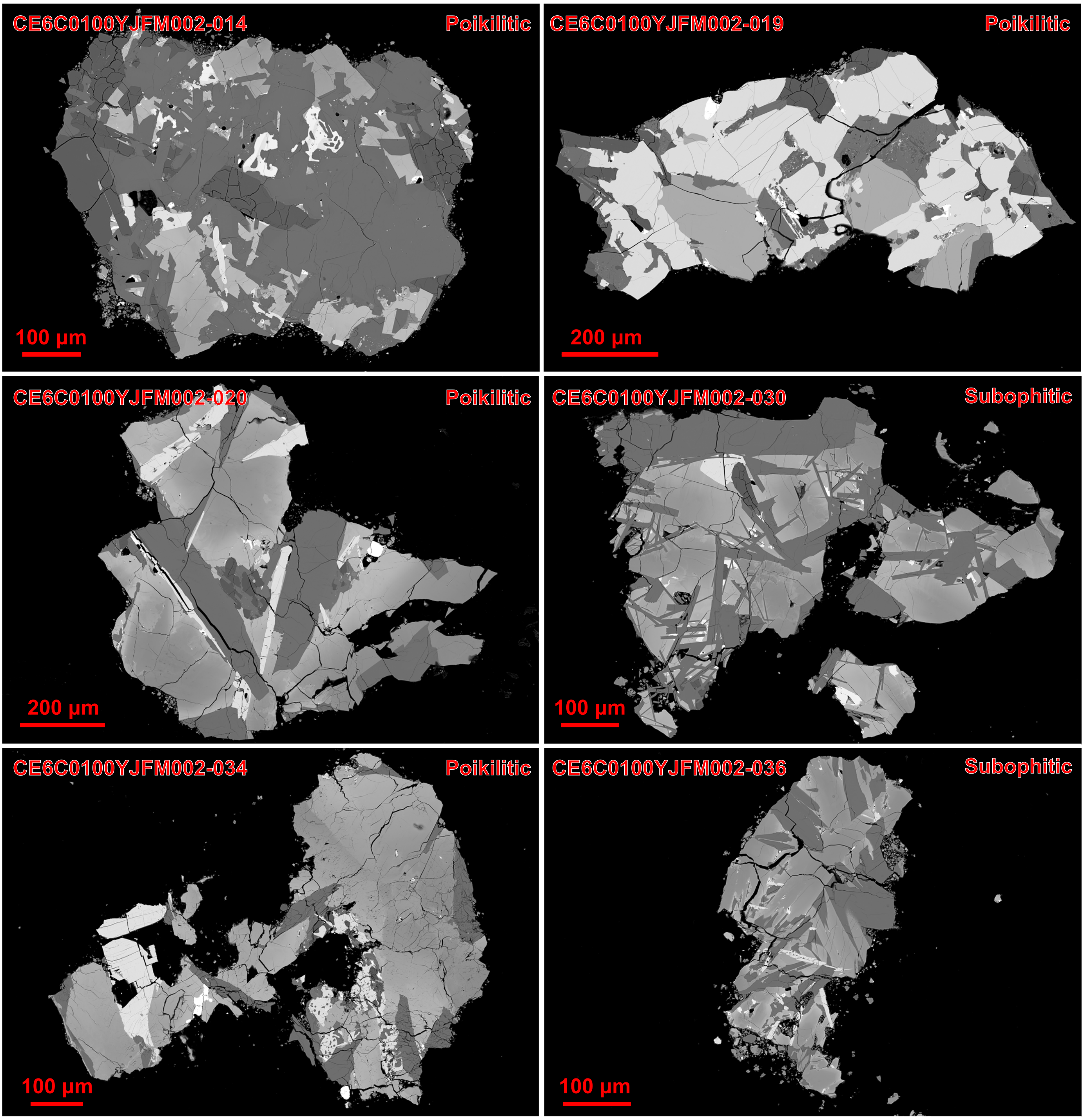
CE6C0100YJFM002-014
Poikilitic
100 μm
CE6C0100YJFM002-019
Poikilitic
200 μm
CE6C0100YJFM002-020
Poikilitic
200 μm
CE6C0100YJFM002-030
Subophitic
100 μm
CE6C0100YJFM002-034
Poikilitic
100 μm
CE6C0100YJFM002-036
Subophitic
100 μm

Fig. S1-3

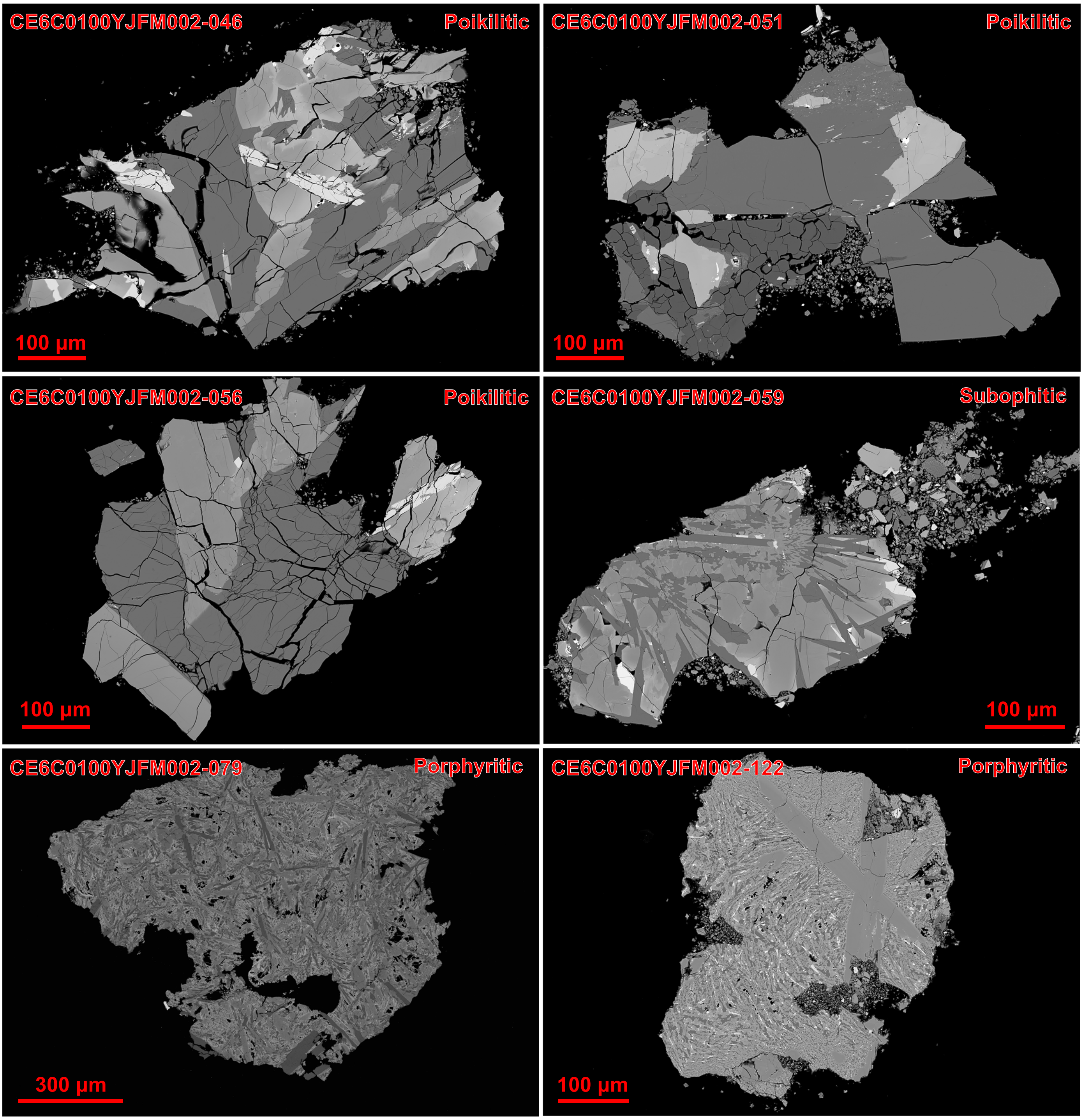
CE6C0100YJFM002-046
Poikilitic
100 µm
CE6C0100YJFM002-051
Poikilitic
100 µm
CE6C0100YJFM002-056
Poikilitic
100 µm
CE6C0100YJFM002-059
Subophitic
100 µm
CE6C0100YJFM002-079
Porphyritic
300 µm
CE6C0100YJFM002-122
Porphyritic
100 µm

Fig. S1-4

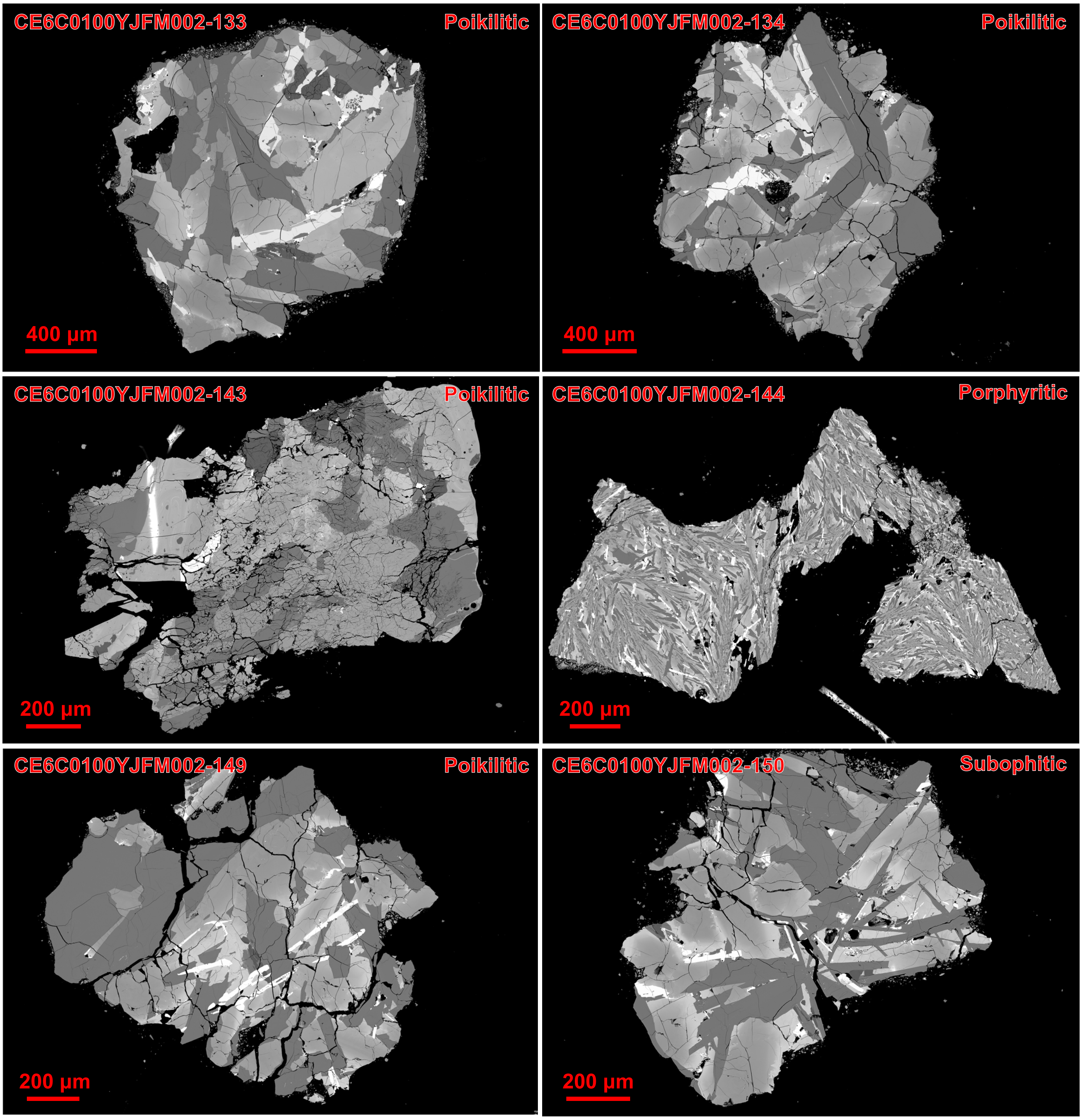
CE6C0100YJFM002-133
Poikilitic
400 μm
CE6C0100YJFM002-134
Poikilitic
400 μm
CE6C0100YJFM002-143
Poikilitic
200 μm
CE6C0100YJFM002-144
Porphyritic
200 μm
CE6C0100YJFM002-149
Poikilitic
200 μm
CE6C0100YJFM002-150
Subophitic
200 μm

Fig. S1-5

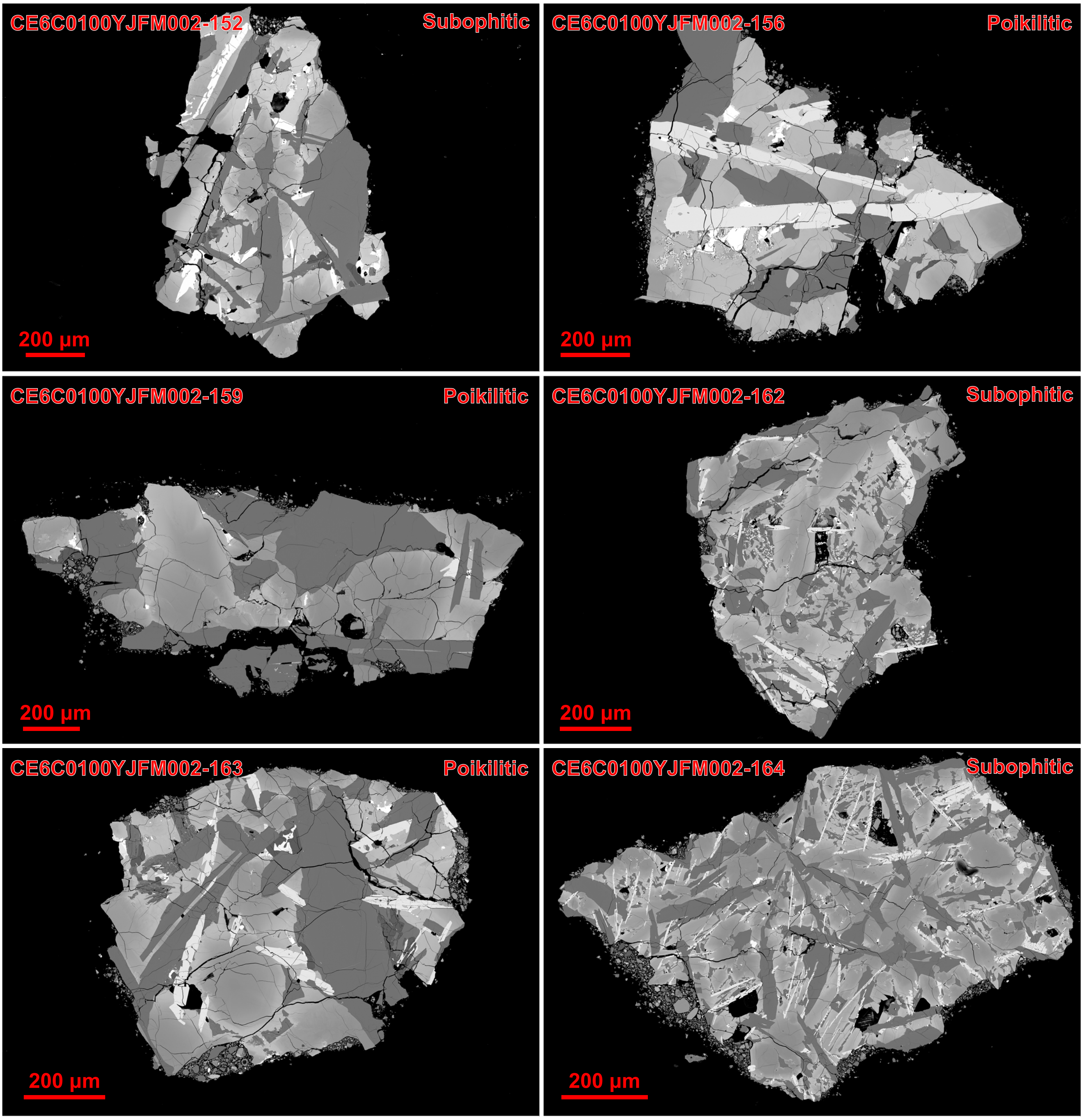
CE6C0100YJFM002-152
Subophitic
200 μm
CE6C0100YJFM002-156
Poikilitic
200 μm
CE6C0100YJFM002-159
Poikilitic
200 μm
CE6C0100YJFM002-162
Subophitic
200 μm
CE6C0100YJFM002-163
Poikilitic
200 μm
CE6C0100YJFM002-164
Subophitic
200 μm

Fig. S1-6

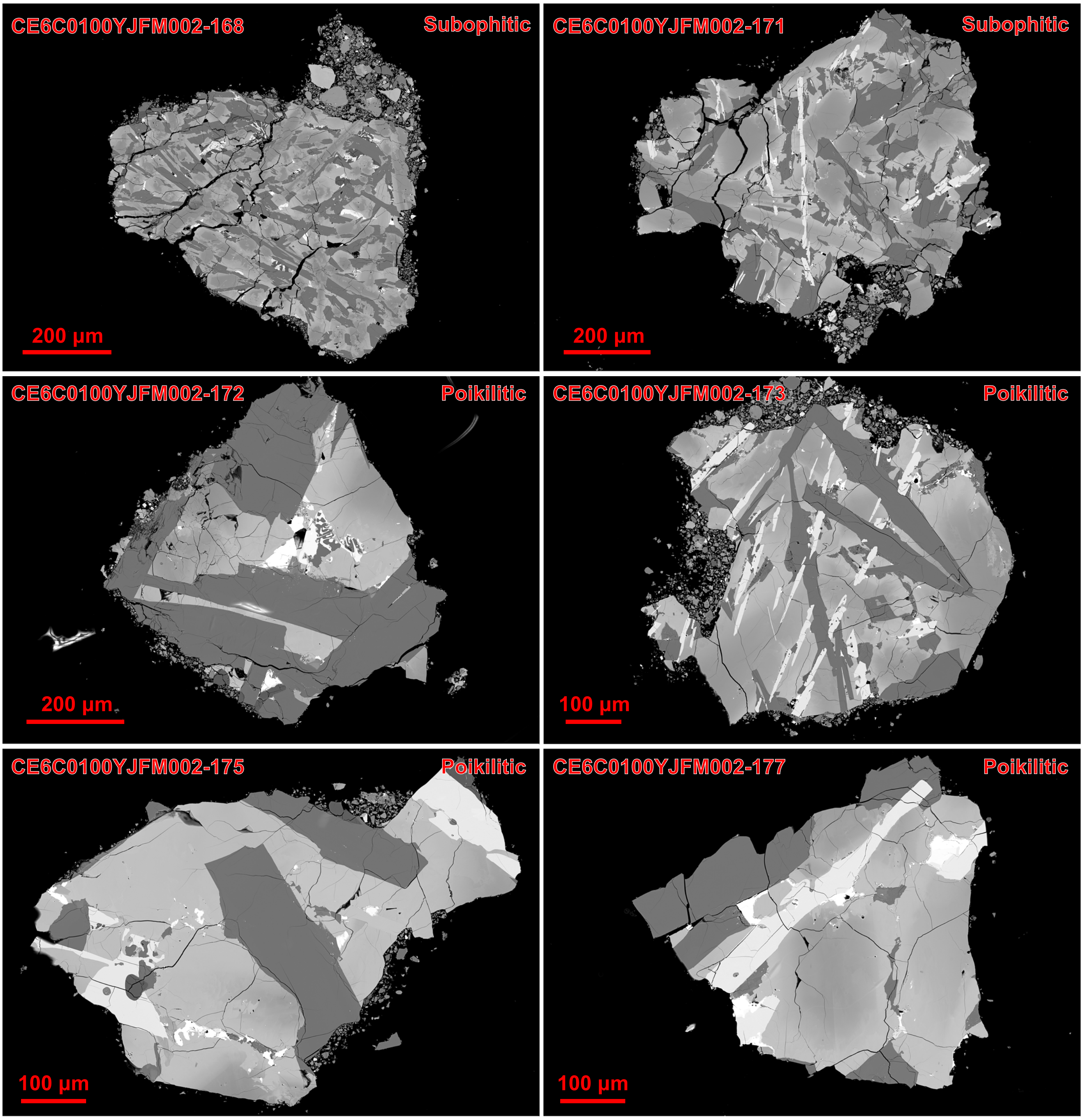
CE6C0100YJFM002-168
Subophitic
200 μm
CE6C0100YJFM002-171
Subophitic
200 μm
CE6C0100YJFM002-172
Poikilitic
200 μm
CE6C0100YJFM002-173
Poikilitic
100 μm
CE6C0100YJFM002-175
Poikilitic
100 μm
CE6C0100YJFM002-177
Poikilitic
100 μm

Fig. S1-7

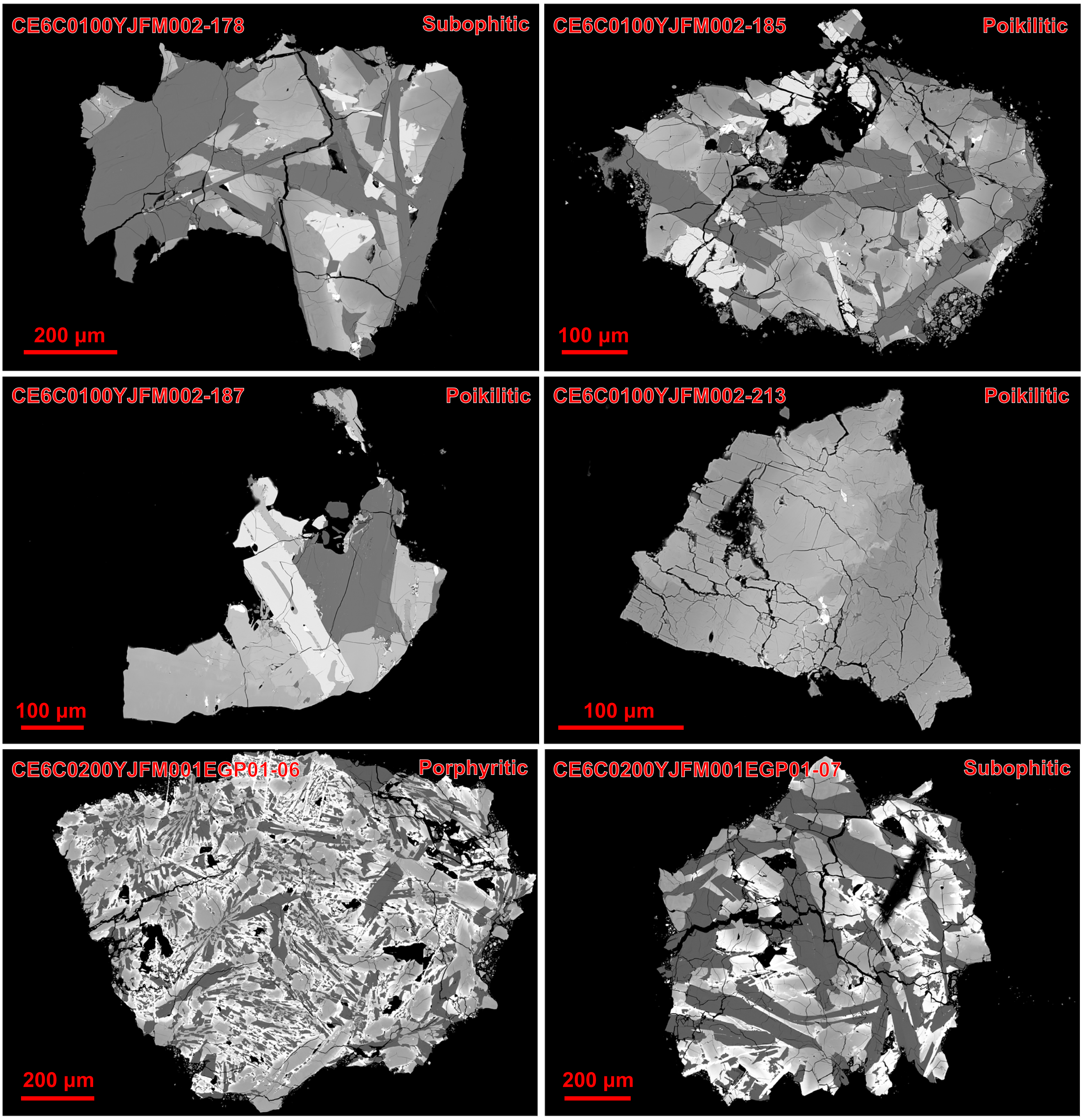
CE6C0100YJFM002-178
Subophitic
200 μm
CE6C0100YJFM002-185
Poikilitic
100 μm
CE6C0100YJFM002-187
Poikilitic
100 μm
CE6C0100YJFM002-213
Poikilitic
100 μm
CE6C0200YJFM001EGP01-06
Porphyritic
200 μm
CE6C0200YJFM001EGP01-07
Subophitic
200 μm

Fig. S1-8

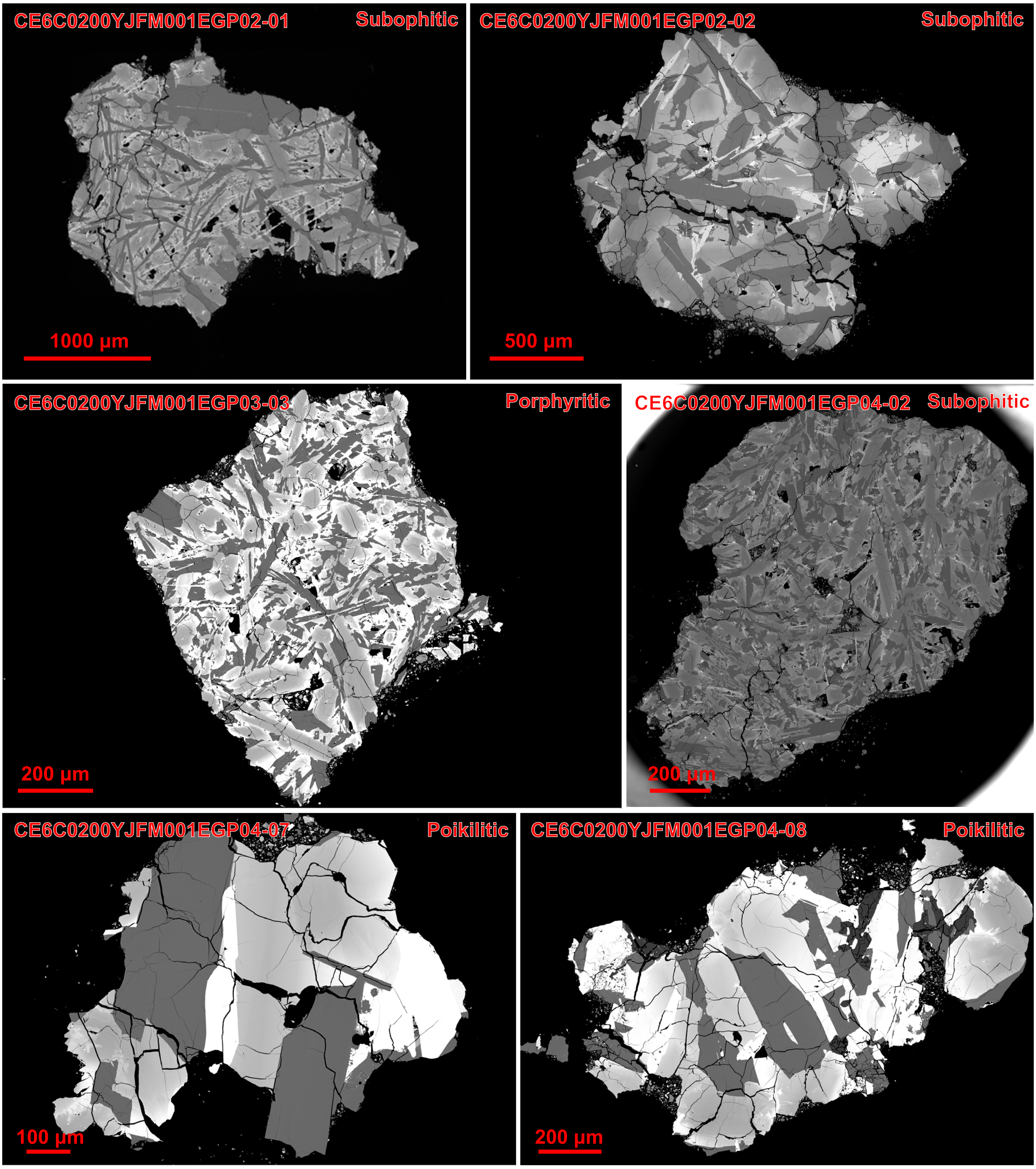
CE6C0200YJFM001EGP02-01
Subophitic
1000 μm
CE6C0200YJFM001EGP02-02
Subophitic
500 μm
CE6C0200YJFM001EGP03-03
Porphyritic
200 μm
CE6C0200YJFM001EGP04-02
Subophitic
200 μm
CE6C0200YJFM001EGP04-07
Poikilitic
100 μm
CE6C0200YJFM001EGP04-08
Poikilitic
200 μm

Fig. S1-9

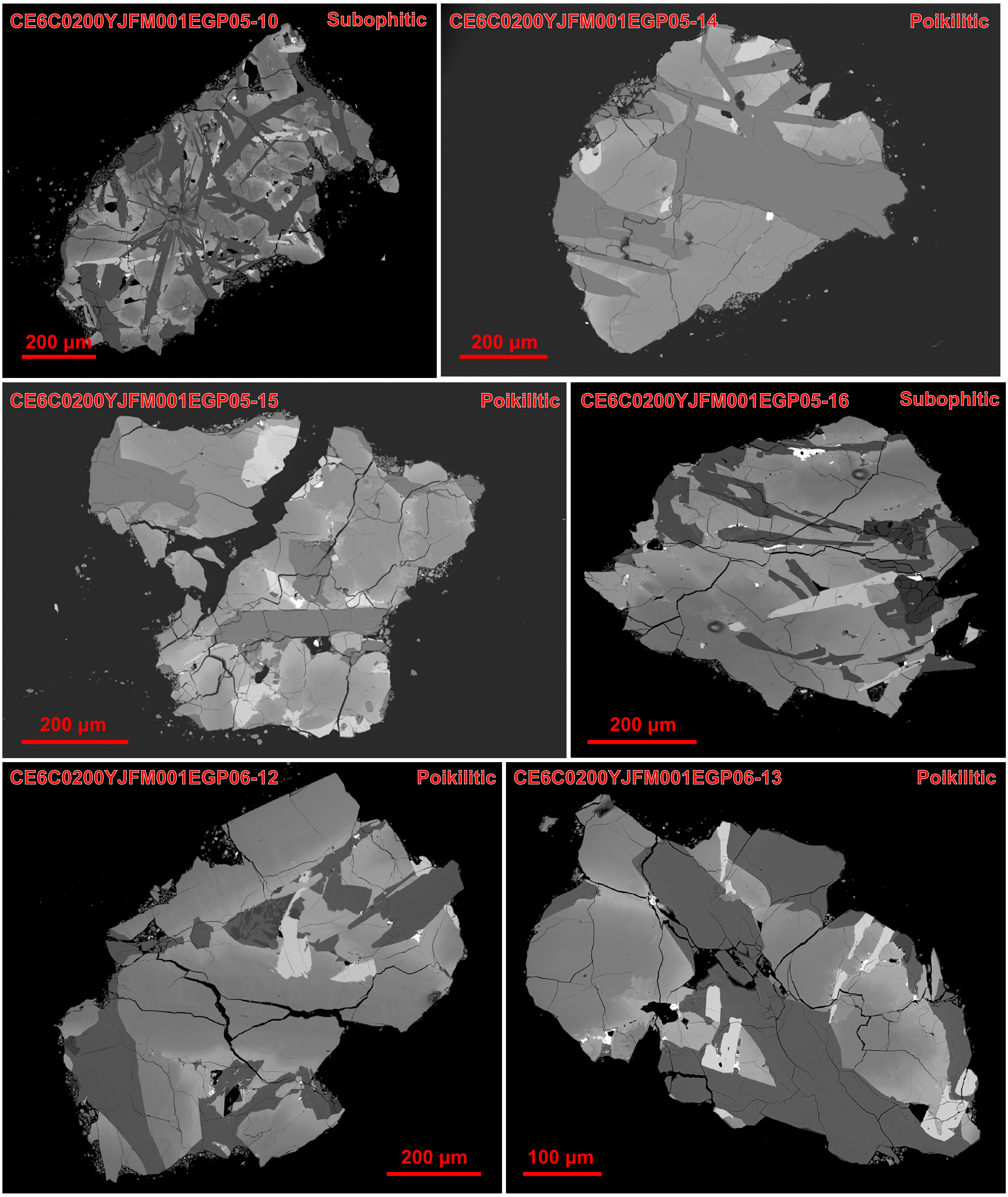
CE6C0200YJFM001EGP05-10
Subophitic
200 µm
CE6C0200YJFM001EGP05-14
Poikilitic
200 µm
CE6C0200YJFM001EGP05-15
Poikilitic
200 µm
CE6C0200YJFM001EGP05-16
Subophitic
200 µm
CE6C0200YJFM001EGP06-12
Poikilitic
200 µm
CE6C0200YJFM001EGP06-13
Poikilitic
100 µm

Fig. S1-10

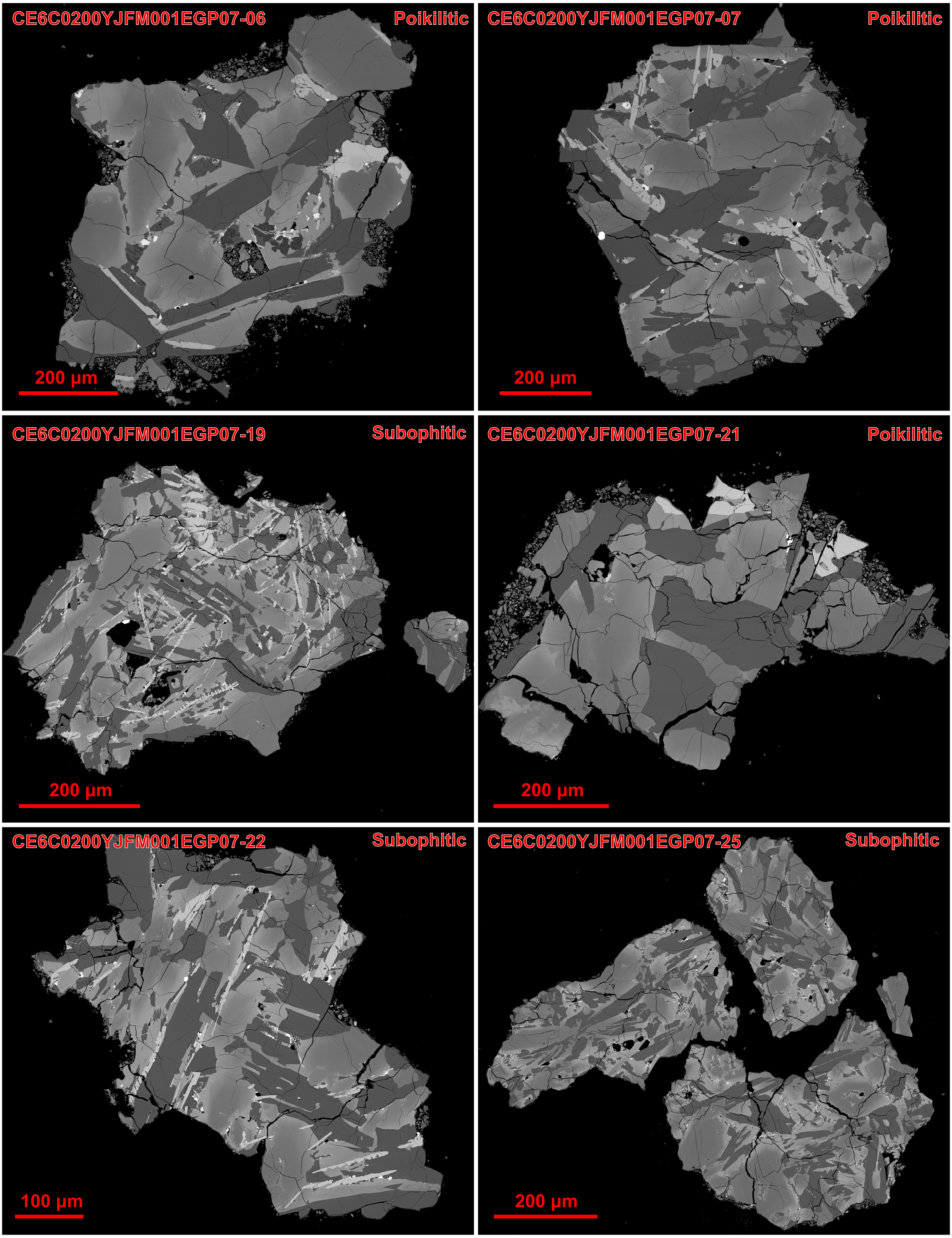
CE6C0200YJFM001EGP07-06
Poikilitic
200 μm
CE6C0200YJFM001EGP07-07
Poikilitic
200 μm
CE6C0200YJFM001EGP07-19
Subophitic
200 μm
CE6C0200YJFM001EGP07-21
Poikilitic
200 μm
CE6C0200YJFM001EGP07-22
Subophitic
100 μm
CE6C0200YJFM001EGP07-25
Subophitic
200 μm

Fig. S1-11

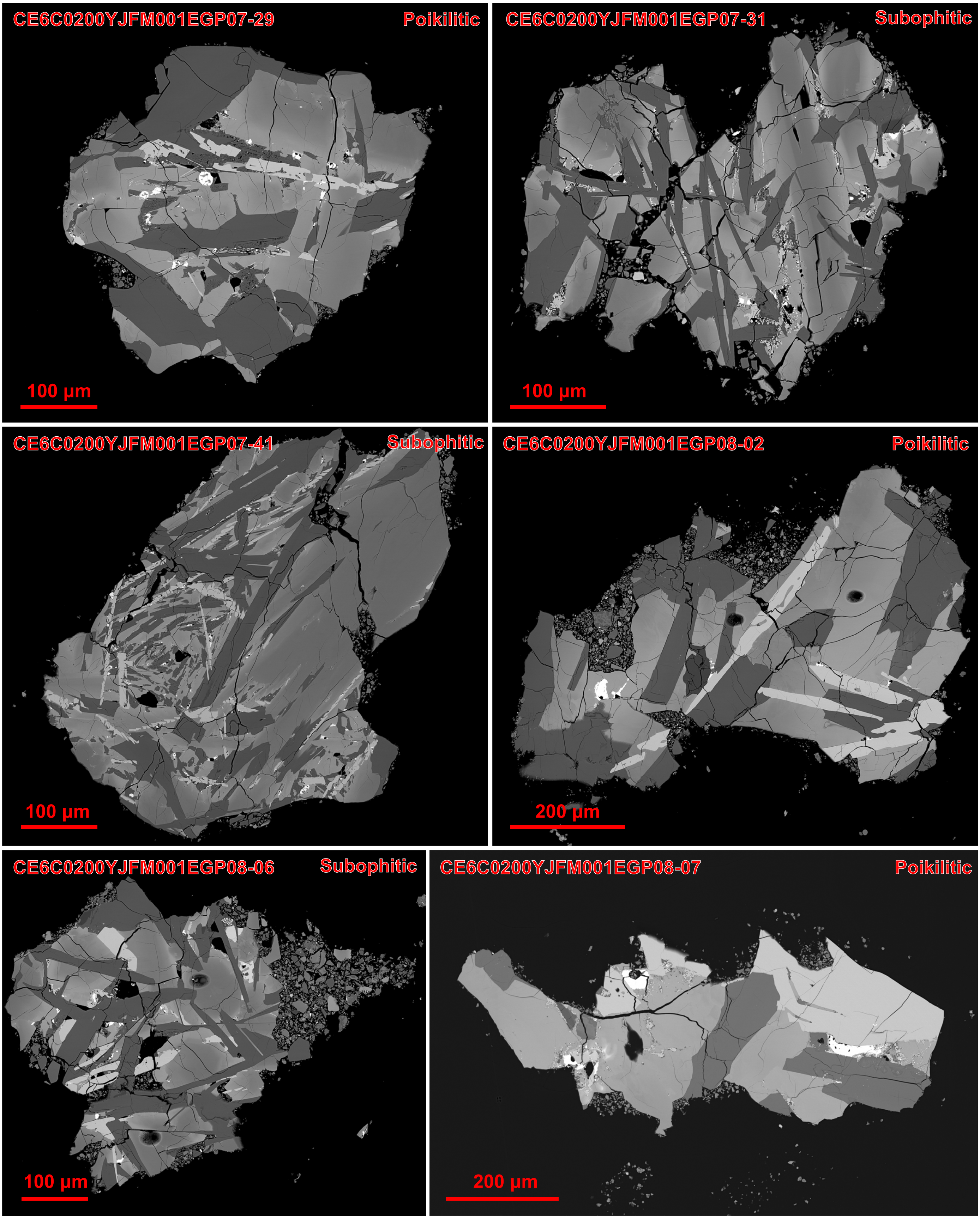

CE6C0200YJFM001EGP07-29
Poikilitic
100 μm
CE6C0200YJFM001EGP07-31
Subophitic
100 μm
CE6C0200YJFM001EGP07-41
Subophitic
100 μm
CE6C0200YJFM001EGP08-02
Poikilitic
200 μm
CE6C0200YJFM001EGP08-06
Subophitic
100 μm
CE6C0200YJFM001EGP08-07
Poikilitic
200 μm

Fig. S1-12

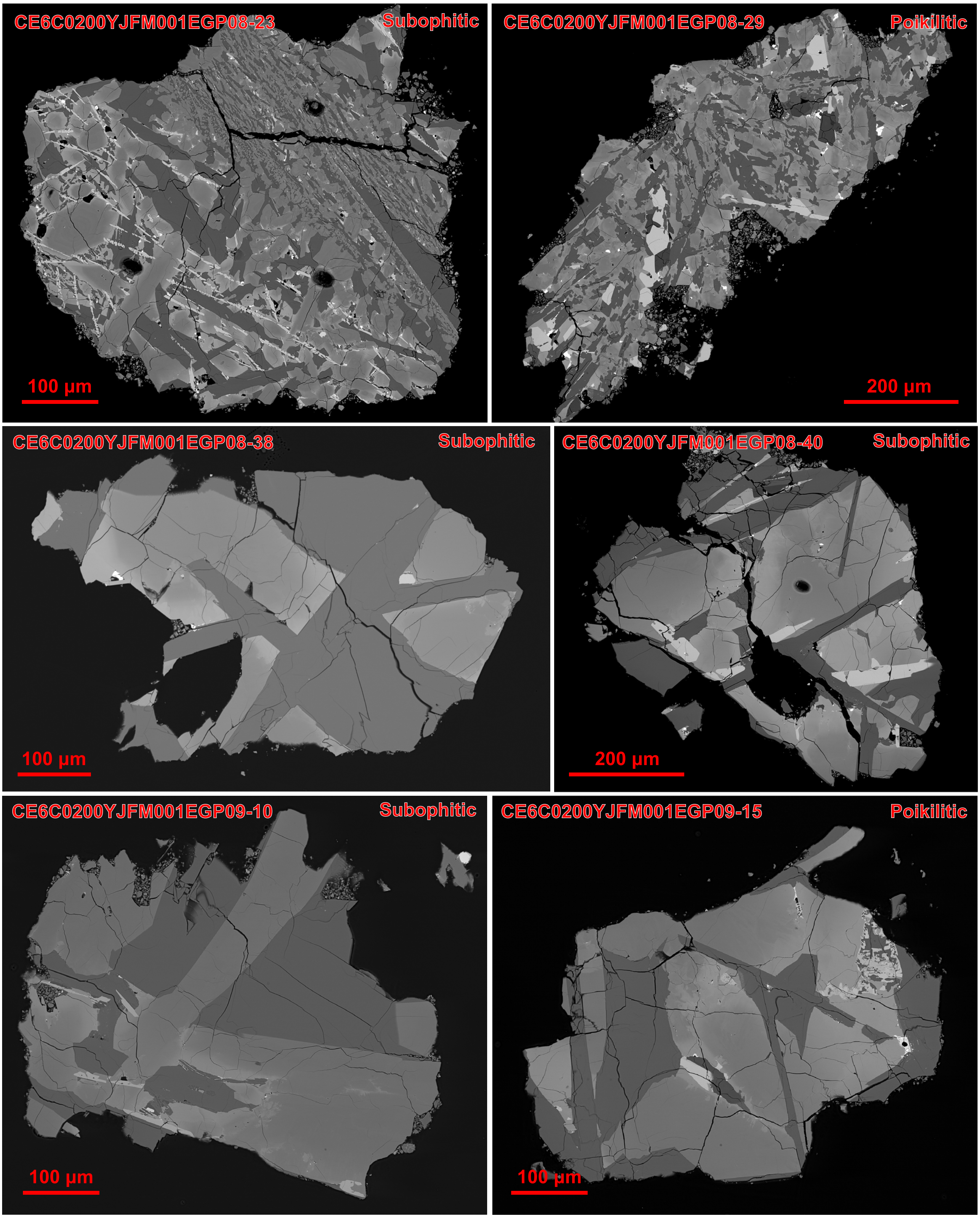
CE6C0200YJFM001EGP08-23
Subophitic
100 μm
CE6C0200YJFM001EGP08-29
Poikilitic
200 μm
CE6C0200YJFM001EGP08-38
Subophitic
100 μm
CE6C0200YJFM001EGP08-40
Subophitic
200 μm
CE6C0200YJFM001EGP09-10
Subophitic
100 μm
CE6C0200YJFM001EGP09-15
Poikilitic
100 μm

Fig. S1-13

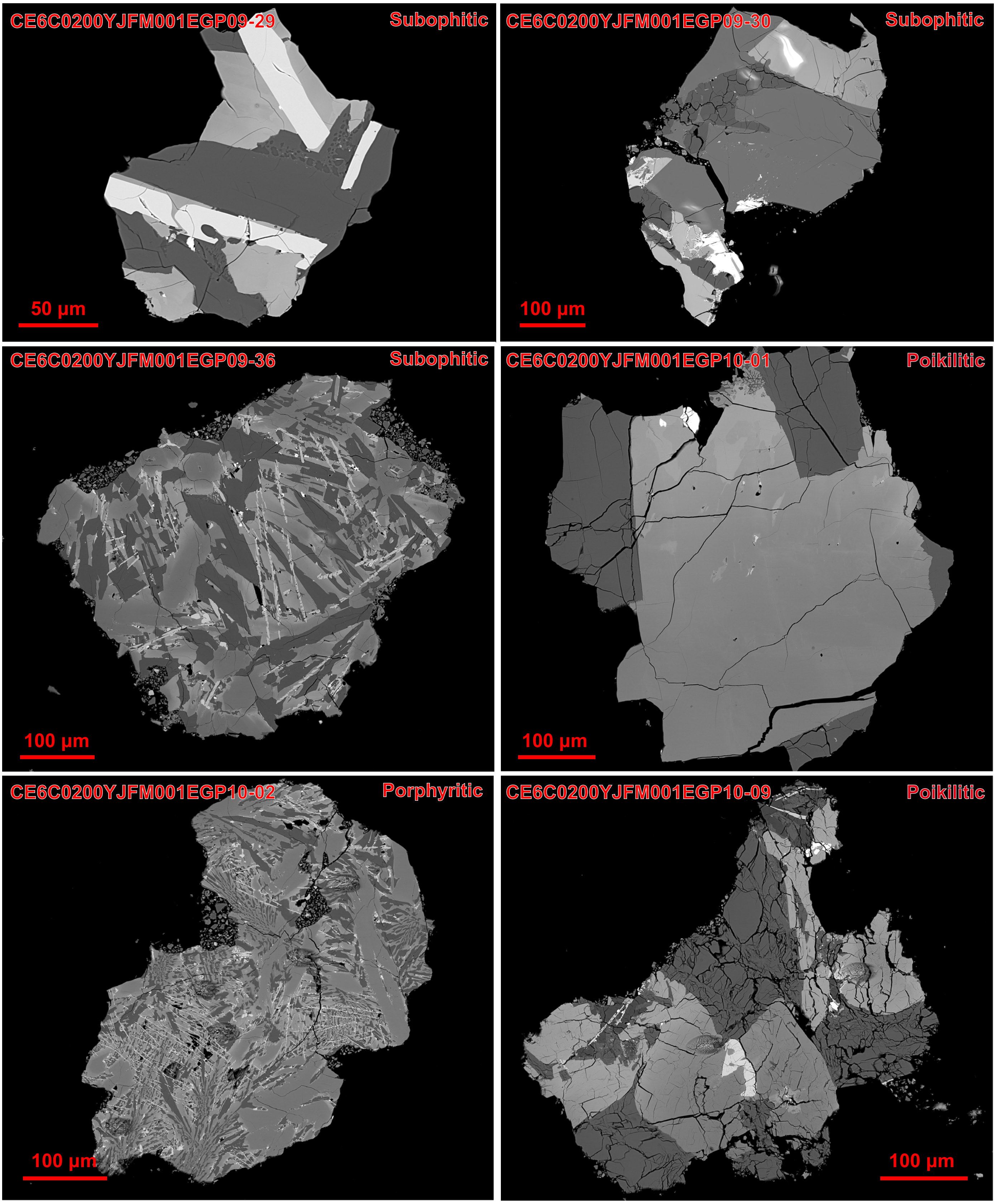
CE6C0200YJFM001EGP09-29
Subophitic
50 μm
CE6C0200YJFM001EGP09-30
Subophitic
100 μm
CE6C0200YJFM001EGP09-36
Subophitic
100 μm
CE6C0200YJFM001EGP10-01
Poikilitic
100 μm
CE6C0200YJFM001EGP10-02
Porphyritic
100 μm
CE6C0200YJFM001EGP10-09
Poikilitic
100 μm

Fig. S1-14

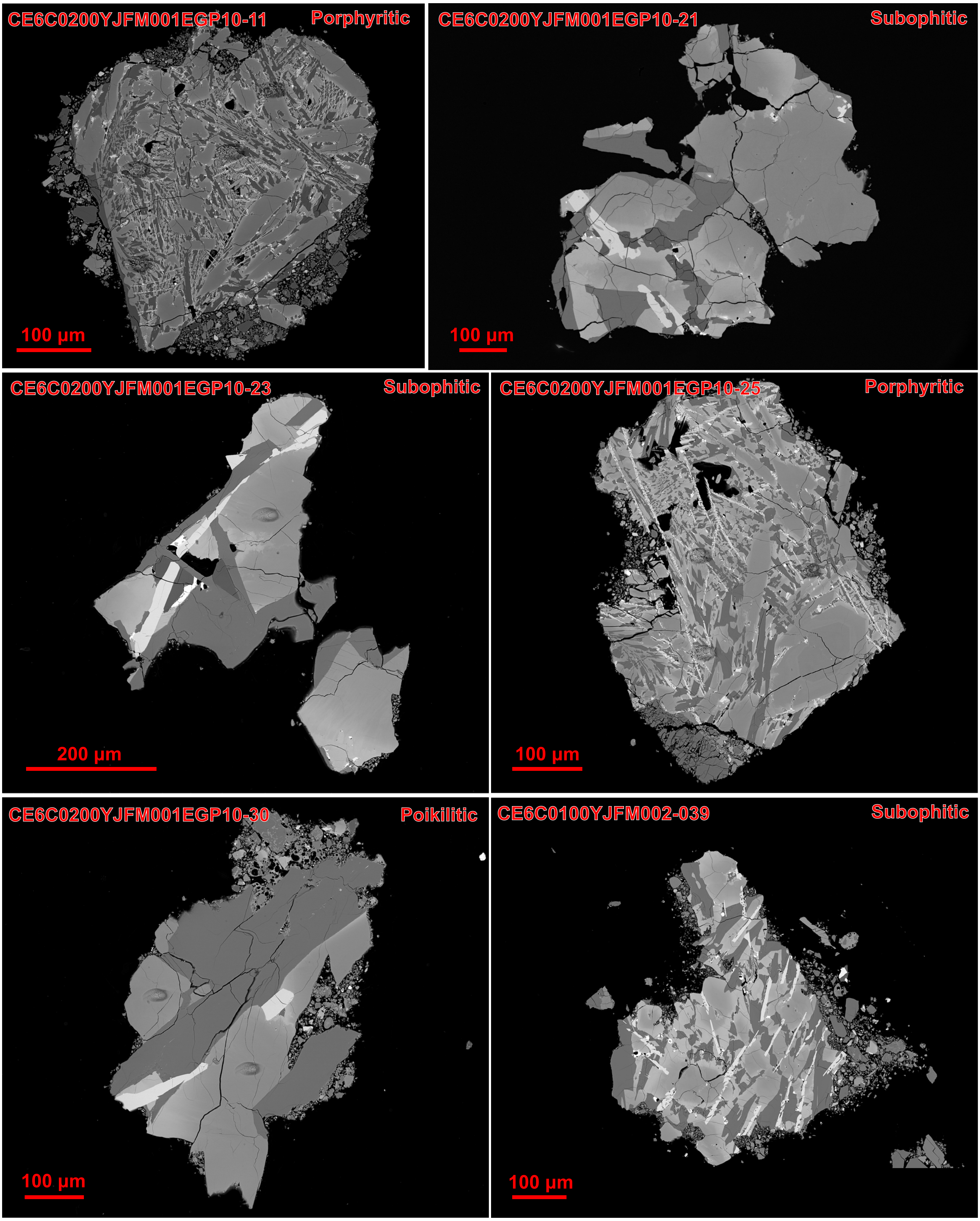
CE6C0200YJFM001EGP10-11
Porphyritic
100 μm
CE6C0200YJFM001EGP10-21
Subophitic
100 μm
CE6C0200YJFM001EGP10-23
Subophitic
200 μm
CE6C0200YJFM001EGP10-25
Porphyritic
100 μm
CE6C0200YJFM001EGP10-30
Poikilitic
100 μm
CE6C0100YJFM002-039
Subophitic
100 μm

Fig. S1-15

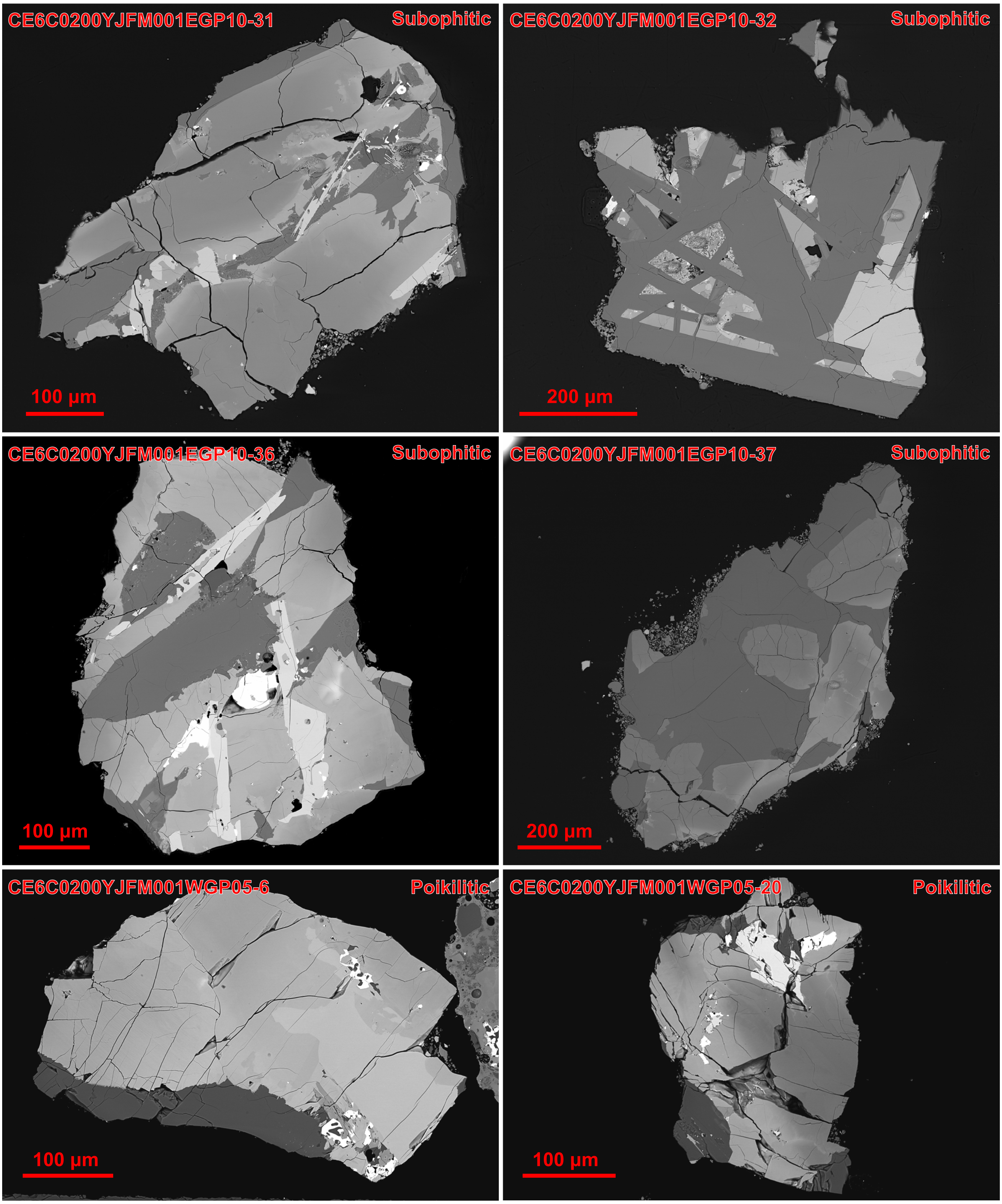
CE6C0200YJFM001EGP10-31
Subophitic
100 μm
CE6C0200YJFM001EGP10-32
Subophitic
200 μm
CE6C0200YJFM001EGP10-36
Subophitic
100 μm
CE6C0200YJFM001EGP10-37
Subophitic
200 μm
CE6C0200YJFM001WGP05-6
Poikilitic
100 μm
CE6C0200YJFM001WGP05-20
Poikilitic
100 μm

Fig. S1-16

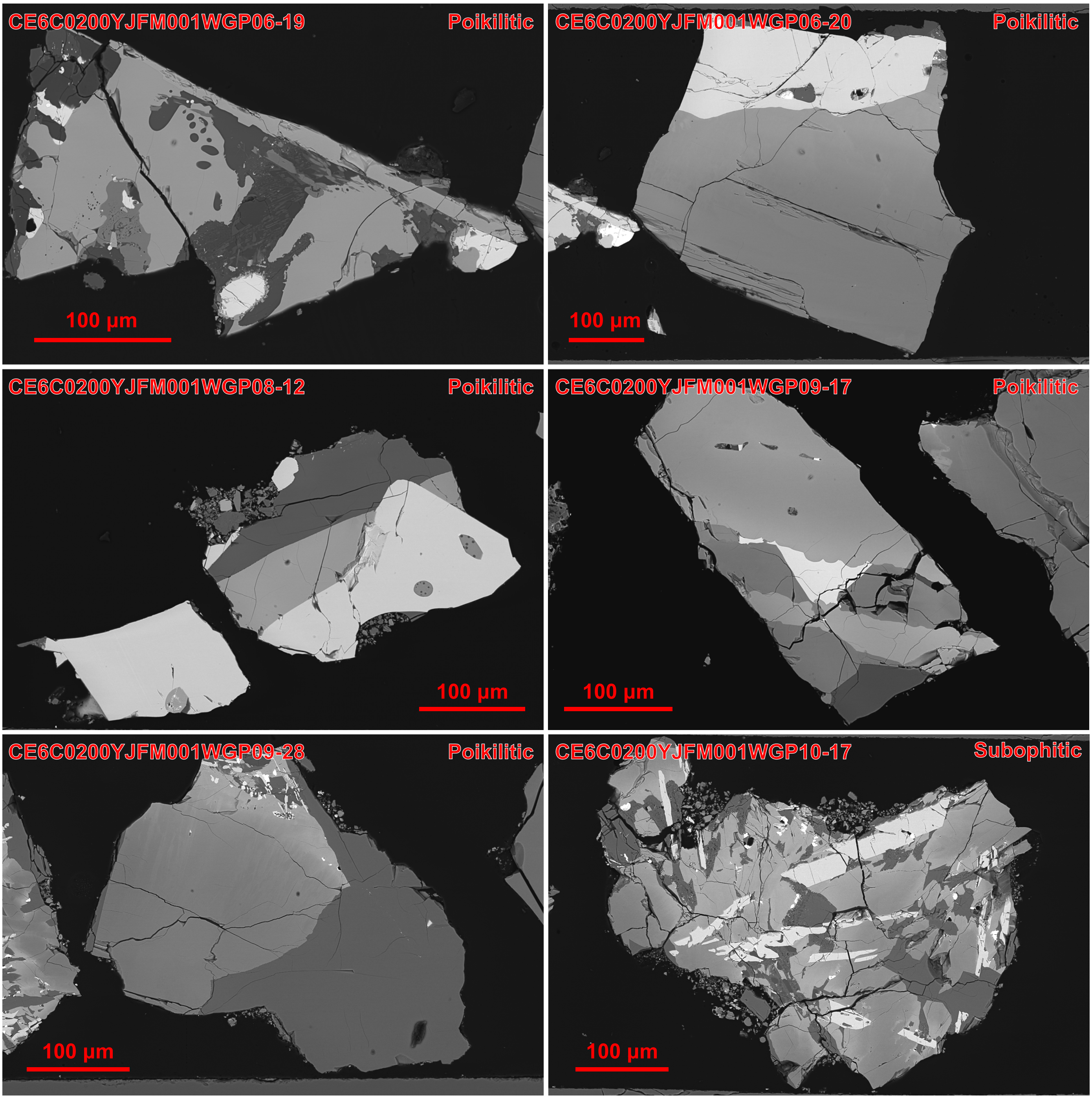
CE6C0200YJFM001WGP06-19
Poikilitic
100 μm
CE6C0200YJFM001WGP06-20
Poikilitic
100 μm
CE6C0200YJFM001WGP08-12
Poikilitic
100 μm
CE6C0200YJFM001WGP09-17
Poikilitic
100 μm
CE6C0200YJFM001WGP09-28
Poikilitic
100 μm
CE6C0200YJFM001WGP10-17
Subophitic
100 μm

Fig. S1-17

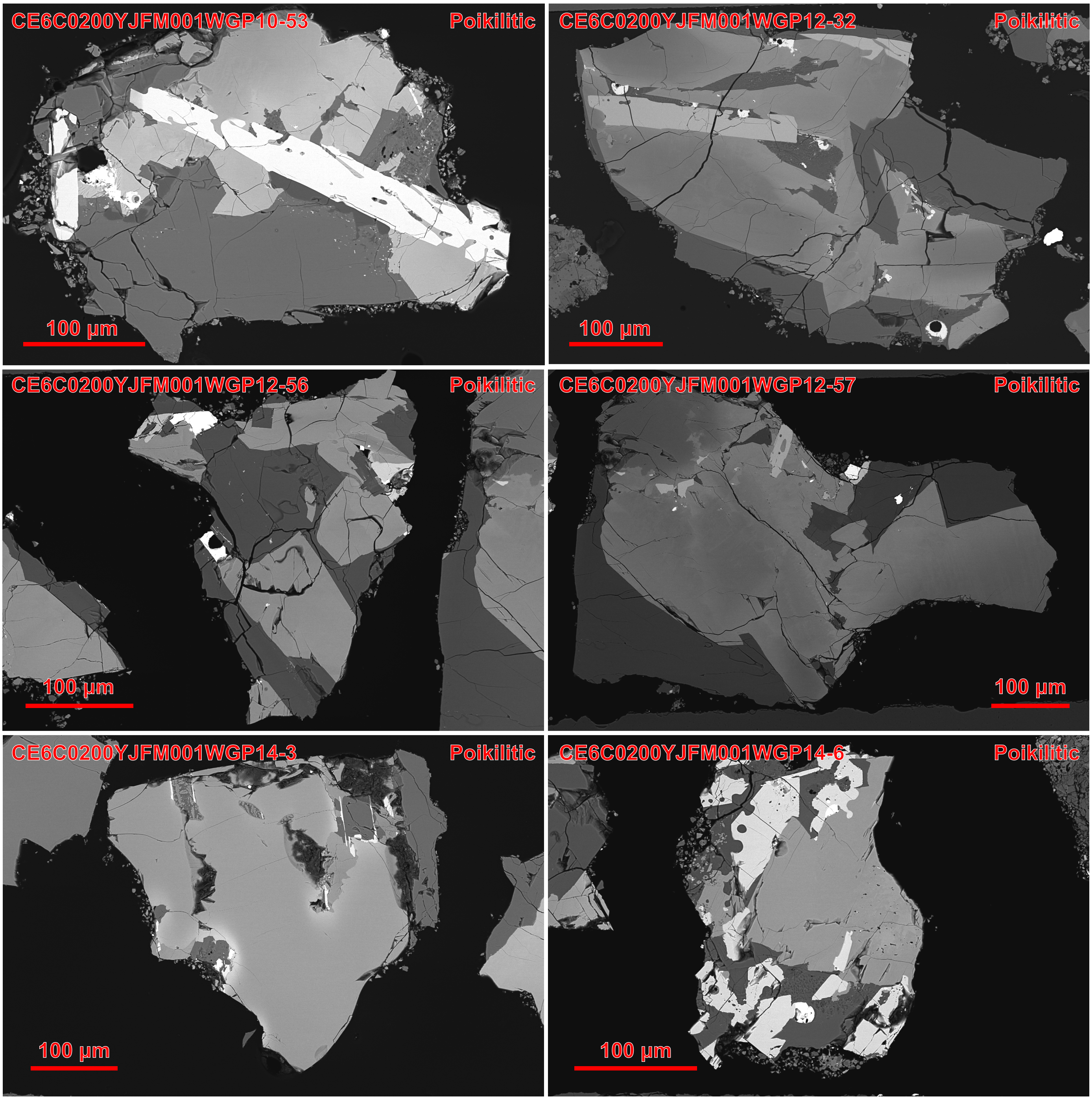
CE6C0200YJFM001WGP10-53
Poikilitic
100 μm
CE6C0200YJFM001WGP12-32
Poikilitic
100 μm
CE6C0200YJFM001WGP12-56
Poikilitic
100 μm
CE6C0200YJFM001WGP12-57
Poikilitic
100 μm
CE6C0200YJFM001WGP14-3
Poikilitic
100 μm
CE6C0200YJFM001WGP14-6
Poikilitic
100 μm

Fig. S1-18
